# Molecular Engineering toward High‐Crystallinity Yet High‐Surface‐Area Porous Carbon Nanosheets for Enhanced Electrocatalytic Oxygen Reduction

**DOI:** 10.1002/advs.202103477

**Published:** 2021-11-16

**Authors:** Yongqi Chen, Junlong Huang, Zirun Chen, Chenguang Shi, Haozhen Yang, Youchen Tang, Zongheng Cen, Shaohong Liu, Ruowen Fu, Dingcai Wu

**Affiliations:** ^1^ PCFM Lab School of Chemistry Sun Yat‐sen University Guangzhou 510275 P. R. China

**Keywords:** carbon nanosheets, electrocatalysts, graphitization, oxygen reduction, porous structure

## Abstract

Carbon‐based nanomaterials have been regarded as promising non‐noble metal catalysts for renewable energy conversion system (e.g., fuel cells and metal–air batteries). In general, graphitic skeleton and porous structure are both critical for the performances of carbon‐based catalysts. However, the pursuit of high surface area while maintaining high graphitization degree remains an arduous challenge because of the trade‐off relationship between these two key characteristics. Herein, a simple yet efficient approach is demonstrated to fabricate a class of 2D N‐doped graphitized porous carbon nanosheets (GPCNSs) featuring both high crystallinity and high specific surface area by utilizing amine aromatic organoalkoxysilane as an all‐in‐one precursor and FeCl_3_·6H_2_O as an active salt template. The highly porous structure of the as‐obtained GPCNSs is mainly attributed to the alkoxysilane‐derived SiO*_x_* nanodomains that function as micro/mesopore templates; meanwhile, the highly crystalline graphitic skeleton is synergistically contributed by the aromatic nucleus of the precursor and FeCl_3_·6H_2_O. The unusual integration of graphitic skeleton with porous structure endows GPCNSs with superior catalytic activity and long‐term stability when used as electrocatalysts for oxygen reduction reaction and Zn–air batteries. These findings will shed new light on the facile fabrication of highly porous carbon materials with desired graphitic structure for numerous applications.

## Graphical abstract

2D N‐doped graphitized porous carbon nanosheets (GPCNSs) featuring both high crystallinity and high specific surface area are prepared by molecular engineering. The unusual integration of graphitic skeleton with porous structure endows GPCNSs with superior catalytic activity and long‐term stability when used as electrocatalysts for oxygen reduction reaction and Zn–air batteries.

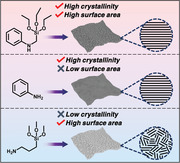

## 1 Introduction

Eco‐friendly, zero emission, and sustainable electrochemical energy conversion technologies, such as fuel cells and rechargeable metal–air batteries, have been considered as promising candidates for the replacement of fossil fuels that suffer from gradually exhaustion and serious environmental issues.^[^ ^1^ ^]^ The performances of these renewable energy devices strongly depend on their indispensable gas‐involving electrochemical redox reactions, such as oxygen reduction reaction (ORR), oxygen evolution reaction (OER), and hydrogen evolution reaction (HER), which are, however, impeded by intrinsically sluggish kinetics and high overpotential.^[^ ^2^ ^]^ Although noble metals (e.g., Pt for ORR and RuO_2_ for OER) have been recognized as peerless catalysts for the electrochemical reactions, the scarce source, high price, and poor durability vastly hamper their large‐scale commercialization.^[^ ^3^ ^]^ Therefore, exploration of cost‐effective non‐noble metal catalysts to fulfill the requirements of practical application is pivotally important for the aforementioned renewable energy conversion system.^[^ ^4^ ^]^

Among the numerous nonprecious metal electrocatalysts, carbon‐based nanomaterials have been extensively exploited and regarded as promising alternatives because of their advantageous properties including low cost, high surface areas, good electrical conductivity, and superior stability.^[^ ^5^ ^]^ In general, the carbon‐based electrocatalysts with optimal activity should have the following merits: i) abundant active sites that enable efficient adsorption of reactants and accelerate the subsequent conversion process;^[^ ^6^ ^]^ ii) high specific surface area, especially with an abundance of mesopores that allow preferable electrolyte wetting, facile mass transport, and sufficient exposure of active sites;^[^ ^7^ ^]^ iii) high degree of graphitization with carbon atoms arranged in long‐range order for fast electron transfer to facilitate the reaction kinetics at the electrode‐electrolyte‐gas triple phase interface.^[^ ^8^ ^]^

Currently, extensive efforts have been devoted to promoting the intrinsic activity and concentration of active sites in carbon skeleton, including heteroatom doping (B, N, P, S, etc.),^[^ ^9^ ^]^ defect engineering (topological defects, edge defects, vacancies, etc.),^[^ ^10^ ^]^ and atomically dispersed transition metal on nitrogen doped carbon (M—N—C, M═Fe, Co, Ni, Cu, Mn, etc.).^[^ ^11^ ^]^ Although their catalytic performances have been demonstrated to be effectively enhanced, there remains a notorious tradeoff for the carbon‐based catalysts between their graphitization degree and specific surface area. On one hand, carbon materials with high crystallinity are usually generated by high‐temperature/catalytic graphitization process, during which enormous carbon atoms are rearranged to form long‐range order structure, fatally resulting in the collapse of porous structure and the reduction of surface area.^[^ ^12^ ^]^ On the other hand, the carbon materials with well‐developed porosity are generally fabricated by post activation or template methods, accompanied with the decrease of crystallite size and formation of numerous structural defects, thereby leading to a dominated amorphous structure with poor electrical conductivity.^[^ ^13^ ^]^ Considering that both graphitic skeleton and porous structure are critical for the performances of carbon‐based catalysts, it is urgently imperative but still challenging to unblock the trade‐off relationship between these two characteristics, and thereby, to promote practical applications of carbon‐based catalysts in renewable energy conversion systems.

Herein, we report a simple yet efficient approach for the fabrication of 2D N‐doped graphitized porous carbon nanosheets (GPCNSs) featuring both high crystallinity and high specific surface area. Key to this novel preparation strategy is the elaborate employment of amine aromatic organoalkoxysilane (e.g., anilino‐methyl‐triethoxysilane, AMS) as an all‐in‐one precursor and FeCl_3_·6H_2_O as an active salt template. Specifically, the aromatic nucleus in AMS is favorable for the formation of highly graphitized structure during catalytic pyrolysis process enabled by FeCl_3_·6H_2_O salt; while the alkoxysilane group can be easily crosslinked via hydrolysis reaction and in situ transformed into ultrafine SiO*_x_* nanoclusters after carbonization, which can function as micro/mesopore templates to significantly increase the surface area of GPCNSs; in addition, the amine group enables the formation of N‐doped carbon skeleton with sufficient localized reactive sites. Benefiting from the synergistic merits, the as‐obtained GPCNSs deliver appealing catalytic activity along with high methanol tolerance and long‐term stability toward ORR, outperforming the benchmark Pt/C catalyst. Moreover, a primary home‐made Zn–air battery further reveals the potential and superior performance of GPCNSs electrocatalyst. These interesting findings will shed new light on the facile fabrication of graphitized porous carbon materials and boost their performances in various applications.

## 2 Results and Discussion

**Figure 1:**
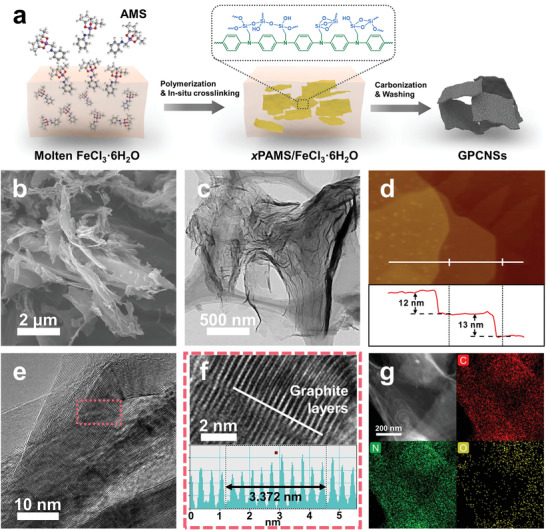
a) Schematic illustration of the synthetic procedure of GPCNSs. The second graphitization process after washing steps is not shown. b) SEM, c) TEM, d) AFM, and e,f) HRTEM images of GPCNSs. g) HAADF‐STEM image and the corresponding EDS element mappings showing the dispersion of C, N, and O elements in GPCNSs. The inset in f) is the intensity profiles along the white line illustrating lattice spacing.

The synthetic approach of GPCNSs is schematically depicted in **Figure** **1**a. 2D crosslinked polyAMS (*x*PAMS) is first obtained by directly mixing AMS molecules with molten FeCl_3_·6H_2_O that is applied as a 2D active salt template (Figure S1, Supporting Information).^[^ ^14^ ^]^ During this process, the triethoxysilane group of AMS is hydrolyzed and condensed to form crosslinked polysilsesquioxane network, while the anilino group undergoes oxidative polymerization under the catalysis of Fe^3+^ (Figure S2a, Supporting Information). Fourier‐transform infrared (FT‐IR) and ^13^C magic angle spinning nuclear magnetic resonance (^13^C MAS‐NMR) measurements are further taken to investigate the molecular structure of *x*PAMS. In comparison with AMS, the absence of the stretching vibration band of —NH— group (3417 cm^−1^) in the spectrum of *x*PAMS implies the successful polymerization of anilino groups (Figure S2b, Supporting Information).^[^ ^15^ ^]^ Meanwhile, a new broad peak arises at 1050–1180 cm^−1^ that can be attributed to the asymmetric stretching vibration of Si—O—Si group, accompanied by the disappearance of absorption peaks of Si—O—C group at 1080 and 1103 cm^−1^, indicating the formation of polysilsesquioxane networks.^[^ ^16^ ^]^ This is further evidenced by obvious decrease of the relative intensity of sp^3^ C peaks in the ^13^C NMR spectrum of *x*PAMS, revealing that a great deal of ethoxy groups is stripped off during in situ hydrolyzation and condensation of triethoxysilane (Figure S2c, Supporting Information).

The mixture of *x*PAMS and FeCl_3_·6H_2_O salt is then thermally annealed at 700 °C in flowing inert atmosphere, followed by etching and washing to remove the residual iron and silica species. The resulting products are further subjected to graphitization under 1000 °C, giving rise to GPCNSs. The scanning electron microscopy (SEM) and transmission electron microscopy (TEM) images show that GPCNSs possess a lateral size ranging from hundreds of nanometers to several micrometers, indicating the 2D morphology can be well maintained during carbonization process (Figure 1b,c). No metal particles are observed within all the carbon skeleton. The atomic force microscopy (AFM) image in Figure 1d indicates that the sheet‐like GPCNSs have a thickness of ≈12–13 nm. The high‐resolution TEM (HRTEM) image clearly reveals that GPCNSs are composed of long‐range order carbon layers with a typical graphite structure, which results from the catalytic pyrolysis by the Fe species at high temperature (Figure 1e; and Figure S3, Supporting Information). The regular lattice fringes of GPCNSs show a well‐defined interlayer spacing of 0.3372 nm, which can be ascribed to the (002) plane of graphite, implying the high crystallinity of graphitic skeleton (Figure 1f).^[^ ^17^ ^]^ The high‐angle annular dark field scanning TEM (HAADF‐STEM) image and the corresponding energy‐dispersive X‐ray spectroscopy (EDS) element mappings verify that C, N, and O are homogeneously dispersed in GPCNSs (Figure 1g). Owing to the very few amounts of residual Fe (0.38 wt%) and Si (0.07 wt%) according to inductively coupled plasma optical emission spectroscopy (ICP‐OES) analysis, no obvious Fe and Si signals are observed within the framework (Figure S4, Supporting Information). Aberration‐corrected HAADF‐STEM image further confirms the absence of bright dots related to single metal atoms (Figure S5, Supporting Information).

**Figure 2:**
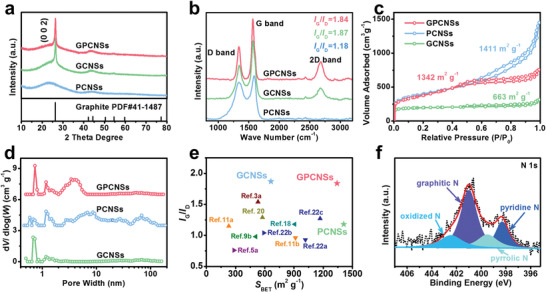
a) XRD patterns, b) Raman spectra, c) N_2_ adsorption–desorption isotherms, and d) pore size distribution curves of GPCNSs, GCNSs and PCNSs. e) Comparison of the *S*_BET_ and *I*_G_/*I*_D_ of GPCNSs, GCNSs, and PCNSs with the those of previously reported carbon nanomaterials. f) High‐resolution N 1s XPS spectrum of GPCNSs.

The structural characteristics of GPCNSs are identified by X‐ray diffraction (XRD) and Raman spectroscopy analysis. The XRD pattern of GPCNSs shows a sharp (002) peak at 26.4°, indicating the highly graphitic layered structure (**Figure** **2**a). The average lattice spacing is calculated to be 0.3375 nm, corresponding to a graphitization index (*g*_p_) as high as 0.76, which is much higher than previous reports including single‐crystal‐like carbon membranes (*g*_p_ = 0.46).^[^ ^18^ ^]^ According to Scherrer equation, the lateral size (*L*_a_) of GPCNSs is calculated to be ≈102.2 nm, while the stacking height of graphite layer (*L*_c_) is ≈15.5 nm, consistent with the AFM analysis.^[^ ^19^ ^]^ The Raman spectrum of GPCNSs in Figure 2b shows a sharp and strong G band (1570 cm^−1^, graphitic structure) and a broad D band (1340 cm^−1^, defective structure).^[^ ^20^ ^]^ Remarkably, the intensity ratio of G‐band to D‐band (*I*_G_/*I*_D_) is up to 1.84 and a sharp 2D band is located at 2680 cm^−1^, confirming the domination of highly crystalline graphitic structure.^[^ ^21^ ^]^ The N_2_ adsorption–desorption measurements are further conducted to analyze the pore characteristics for GPCNSs. As shown in Figure 2c, GPCNSs exhibit a typical type‐IV isotherm with an uptake at low relative pressure and an obvious hysteresis loop, implying the presence of micro‐ and mesopores. The pore size distribution curve demonstrates that GPCNSs have abundant micropores at 0.7 and 1.2 nm, and well‐developed mesopores centered at 4.0 nm (Figure 2d). Notably, the Brunauer–Emmett–Teller (BET) surface area (*S*_BET_) of GPCNSs reaches up to 1342 m^2^ g^−1^ with a dominant external surface area of 1041 m^2^ g^−1^. To the best of our knowledge, carbon nanomaterials with such a high porosity structure and a high degree of graphitization have not been achieved so far (Figure 2e).^[^ ^3^, ^5^, ^9^, ^11^, ^18^, ^20^, ^22^ ^]^ The chemical composition of the GPCNSs is investigated by X‐ray photoelectron spectroscopy (XPS) and reveals the presence of C, N, and O elements in GPCNSs (Figure S6, Supporting Information). The content of N is calculated to be 5.2 wt%. The high‐resolution N 1s spectrum visualizes that the nitrogen‐containing functional groups in GPCNSs can be resolved into four types of N species, including pyridine N (398.3 eV), pyrrolic N (399.5 eV), graphitic N (401.0 eV), and oxidized N (402.5 eV), as shown in Figure 2f.^[^ ^23^ ^]^ It has been experimentally and theoretically demonstrated that these N dopants can create sufficient localized reactive sites and thus effectively promote the reaction kinetics in ORR.^[^ ^24^ ^]^

**Figure 3:**
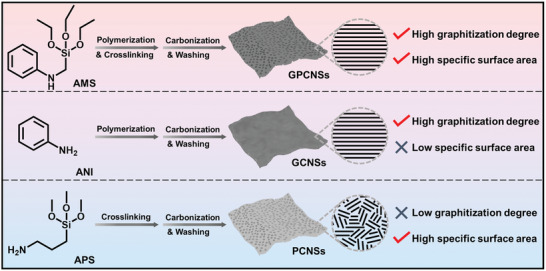
Schematic illustration of the different structures of GPCNSs, GCNSs, and PCNSs obtained from AMS, ANI, and APS precursors, respectively.

To unveil the mechanisms involved in the formation of GPCNSs featuring both high crystallinity and high specific surface area, the roles of FeCl_3_·6H_2_O salt and pyrolysis process are studied. As shown in Figure S7 (Supporting Information), the resulting products obtained by pyrolyzing *x*PAMS without FeCl_3_·6H_2_O salt possess typical characteristics of amorphous carbon, indicating the important role of FeCl_3_·6H_2_O for graphitization. XRD patterns of annealed *x*PAMS/FeCl_3_·6H_2_O mixture without acid washing show that a certain amount of Fe/Fe_3_C is formed when the temperature reaches 700 °C (Figure S8, Supporting Information), which can functionalize as catalysts to promote migration and recrystallization of the carbon atoms into graphite.^[^ ^25^ ^]^ Notably, no Fe/Fe_3_C can be formed at a lower annealing temperature (e.g., 600 °C), leading to the formation of porous carbon nanosheets with an amorphous structure (*I*_G_/*I*_D_ = 1.04) even after graphitization treatment at 1000 °C (Figure S9, Supporting Information). Meanwhile, the in situ formed Fe/Fe_3_C catalyst suffers from severe fusion at a higher annealing temperature (e.g., 800 °C), which can destruct the pore structure and cause a lower specific surface area (605 m^2^ g^−1^) (Figure S10, Supporting Information). Additionally, the influence of molecular structure of precursor on the resulting carbon nanostructures is investigated. As shown in Figures S11 and S12 (Supporting Information), similar 2D nanostructures can be produced when aniline (ANI) and (3‐aminopropyl) trimethoxysilane (APS) are used as carbon precursors, respectively, demonstrating the universality of the salt template method to produce 2D porous carbon structures. The XRD, Raman, and HRTEM analyses reveal that graphitized carbon nanosheets (GCNSs) obtained from ANI precursor exhibit a high‐crystallinity carbon skeleton (e.g., 0.78 for *g*_p_, 1.87 for *I*_G_/*I*_D_), while the *S*_BET_ is only 663 m^2^ g^−1^, much lower than that of GPCNSs. On the contrary, the *S*_BET_ of porous carbon nanosheets (PCNSs) obtained from APS precursor is as high as 1411 m^2^ g^−1^, while PCNSs display a broad XRD peak at 23.4° with an *I*_G_/*I*_D_ of only 1.18, suggestive of an amorphous carbon skeleton (Figure 2a–e; and Table S1, Supporting Information). The results demonstrate that the alkoxysilane group makes a great contribution to the highly porosity structure of GPCNSs and the aromatic nucleus is beneficial for the orderly packing of carbon nanodomains to produce highly crystalline graphitic structure (**Figure** **3**).

**Figure 4:**
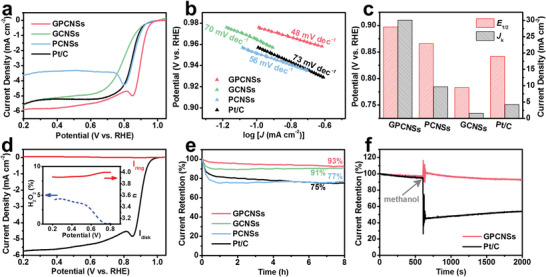
a) LSV curves, b) Tafel plots, and c) the corresponding *E*_1/2_ and *J*_k_ of GPCNSs, GCNSs, PCNSs, and Pt/C catalysts. d) RRDE curves of GPCNSs, and the inset is electron transfer number and peroxide yield. e) Chronoamperometric responses of GPCNSs, GCNSs, PCNSs, and Pt/C catalysts at 0.564 V. f) Chronoamperometric response for ORR at GPCNSs and Pt/C electrodes on addition of 10 mL methanol after about 600 s.

The electrocatalytic performance of GPCNSs toward ORR is first identified by cyclic voltammetry (CV) tests conducted in N_2_ or O_2_‐saturated 0.1 m KOH electrolyte at a scan rate of 20 mV s^−1^ (Figure S13, Supporting Information). Compared with the quasirectangular curve in N_2_‐saturated 0.1 m KOH, a substantial reduction peak is observed at around 0.86 V versus reversible hydrogen electrode (vs RHE) in O_2_‐saturated electrolyte, signifying a high ORR catalytic activity of GPCNSs catalyst. Linear sweep voltammograms (LSV) are further performed to investigate the electrocatalytic activity of GPCNSs by using a rotating disk electrode (RDE) in O_2_‐saturated 0.1 m KOH electrolyte at a scan rate of 10 mV s^−1^. As shown in **Figure** **4**a; and Figure S14 (Supporting Information), the GPCNSs display an onset potential (*E*_onset_) of 0.958 V and a half‐wave potential (*E*_1/2_) of 0.897 V, which are superior to the benchmark Pt/C catalyst (*E*_onset_ = 0.933 V, *E*_1/2_ = 0.842 V) and surpass most previously reported carbon‐based ORR catalysts (Table S2, Supporting Information). The broad current plateau from 0.6 to 0.2 V indicates a diffusion‐controlled process related to four‐electron‐dominated ORR pathway. Notably, the GPCNSs catalyst shows strong resistance to the SCN^−^ poison effect in O_2_‐saturated 0.1 m KOH electrolyte, demonstrating the negligible contribution of trace Fe residual for ORR (Figure S15, Supporting Information). To gain in‐depth insight into the structural advantages of GPCNSs, the electrocatalytic performances of GCNSs and PCNSs are analyzed under identical conditions. As expected, the GCNSs and PCNSs deliver comparatively poor activity in terms of higher onset overpotential (0.936 V for GCNSs, 0.934 V for PCNSs) and half‐wave potential (0.783 V for GCNSs, 0.866 V for PCNSs). The superior catalytic activity of GPCNSs toward ORR is further demonstrated by the smallest Tafel slope of 48 mV dec^−1^, much lower than that of GCNSs (70 mV dec^−1^), PCNSs (56 mV dec^−1^), and even Pt/C catalyst (73 mV dec^−1^) (Figure 4b), verifying the key role of high‐crystallinity and high‐surface‐area structure in facilitating the ORR process. As shown in Figures S16 and S17 (Supporting Information), the GPCNSs exhibit both a much higher electrochemically active surface area (ECSA) and a smaller impedance when compared to GCNSs and PCNSs. These results demonstrate that the well‐developed porous structure in GPCNSs is beneficial for sufficient exposure of active sites, while the highly graphited skeleton enables excellent electrical conductivity for fast reaction kinetics.^[^ ^26^ ^]^

The LSV curves of GPCNSs at various rotating rates are measured to further reveal the reaction kinetics of the ORR process. As shown in Figure S18 (Supporting Information), the current densities increase with increasing rotating speeds as a result of the faster mass diffusion at high speeds. The corresponding Koutecky–Levich (K–L) plots of GPCNSs manifest good parallel linearity and show a similar slope to Pt/C at 0.6 V, suggestive of first‐order reaction kinetics for the ORR with respect to dissolved O_2_ concentration. Moreover, the kinetic limiting current density (*J*_k_) of GPCNSs according to the K–L plot is determined to be 30.1 mA cm^−2^ at 0.85 V, which is 20, 3, and 7 times higher than those of GCNSs (1.5 mA cm^−2^), PCNSs (9.7 mA cm^−2^), and Pt/C (4.2 mA cm^−2^), respectively, indicating excellent ORR kinetics in the GPCNSs with a unique high‐crystallinity and high‐surface‐area structure (Figure 4c). Rotating ring‐disk electrode (RRDE) measurements are carried out to determine the yield of hydrogen peroxide (H_2_O_2_). As demonstrated in Figure 4d, the electron transfer number of GPCNSs is up to 3.92–3.99 under the potential range of 0.2–0.8 V, corresponding to a low peroxide yield of below 5%, suggestive of a desirable selectivity for the four‐electron‐transport process with the reduction of O_2_ to H_2_O. The LSV and chronoamperometric measurements further reveal that the GPCNSs exhibit superior long‐term stability and methanol tolerance to benchmark Pt/C catalyst in O_2_‐saturated 0.1 m KOH (Figure 4e,f; and Figure S19, Supporting Information). Notably, more than 90% current retentions are observed for both GPCNSs and GCNSs after chronoamperometric tests for 8 h, while the PCNSs exhibit a fast current decay to 77% under identical conditions. The results demonstrate that the highly graphitic skeleton is beneficial for suppressing inevitable carbon corrosion during ORR process, thereby significantly improving catalytic durability.^[^ ^27^ ^]^

**Figure 5:**
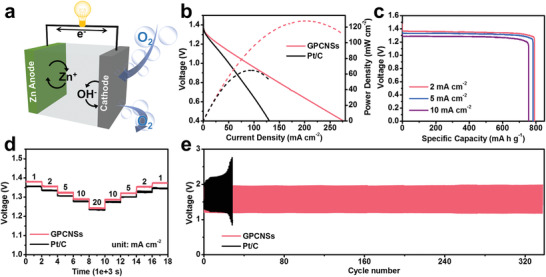
a) Schematic image of a homemade Zn–air battery. b) Discharge curves of Zn–air batteries with GPCNSs and Pt/C catalysts and the corresponding power densities. c) Galvanostatic discharge curves of Zn–air batteries with GPCNSs at different current densities. The specific capacity is normalized by the mass of the consumed Zn anode. d) Discharge profiles at the different current densities (1, 2, 5, 10, 20, and recovery to 1 mA cm^−2^). e) Long‐term cycling tests of the rechargeable Zn–air batteries at *j* = 2 mA cm^−2^ loaded with GPCNSs and Pt/C.

To further investigate the potential application of GPCNSs, a homemade Zn–air battery is assembled by taking GPCNSs‐loaded carbon paper with gas diffusion layer and a zinc foil as cathode and anode, respectively (**Figure** **5**a). As shown in Figure S20 (Supporting Information), the battery with GPCNSs as the air cathode catalyst exhibits a higher open‐circuit voltage of 1.489 V than that with Pt/C catalyst (1.478 V), and two homemade Zn–air batteries with GPCNSs catalyst can be connected in series to easily light up a light‐emitting diode (LED) array panel. According to the polarization curves in Figure 5b, the peak power density of the Zn–air battery with GPCNSs catalyst is 128 mW cm^−2^, largely surpassing that with Pt/C catalyst (65 mW cm^−2^). In addition, the batteries with GPCNSs catalyst can deliver high capacities of 791 and 782 mA h g_Zn_^−1^ at the current densities of 2 and 5 mA cm^−2^, respectively (Figure 5c). These values are higher than that of the batteries with Pt/C catalyst, which exhibit lower capacities of 721 and 716 mA h g_Zn_^−1^ at identical current densities (Figure S21, Supporting Information). Remarkably, a high capacity of 756 mA h g_Zn_^−1^ can still be retained at a high current density of 10 mA cm^−2^ for the battery with GPCNSs catalyst, corresponding to 91% of the theoretical capacity (830 mA h g_Zn_^−1^), suggestive of superior rate capability.^[^ ^28^ ^]^ Moreover, compared to the battery with Pt/C catalyst, obvious smaller discharge voltage drops at various current densities range from 1 to 20 mA cm^−2^ can be observed for the battery with GPCNSs catalyst, verifying accelerated ORR kinetics (Figure 5d). Notably, the primary performance of the Zn–air batteries with GPCNSs catalyst outperform many previously reported carbon‐based ORR catalysts,^[^ ^1^, ^26^, ^29^ ^]^ proving that the synergistic merits of high specific surface area and highly graphitized skeleton are feasible in practical applications (Figure S22 Supporting Information). Considering that GPCNSs also demonstrate good catalytic activity toward OER (Figure S23, Supporting Information), a rechargeable Zn–air battery is further evaluated by galvanostatic charge–discharge at a current density of 2 mA cm^−2^. As shown in Figure 5e, in comparison with the Pt/C‐loaded battery, the battery with GPCNSs catalyst possesses a smaller polarization voltage hysteresis of 0.74 V at initial cycles and superior cycling stability with no obvious voltage fading after 340 cycles, demonstrating the promising application of GPCNSs for the rechargeable Zn–air battery.

## 3 Conclusion

In summary, high‐crystallinity yet high‐surface‐area porous carbon nanosheets have been successfully fabricated with amine aromatic organoalkoxysilane (e.g., AMS) as an all‐in‐one precursor and FeCl_3_·6H_2_O as an active salt template. We have demonstrated that the alkoxysilane group of AMS makes a great contribution to the highly porosity structure of the resulting GPCNSs and the aromatic nucleus of AMS together with the FeCl_3_·6H_2_O salt are synergistically beneficial for the orderly packing of carbon nanodomains to produce highly crystalline graphitic structure. Benefiting from the unusual integration of graphitic skeleton with porous structure, the GPCNSs demonstrate superior catalytic activity and long‐term stability when used as electrocatalysts for ORR and Zn–air batteries. We hope that our present work may provide a universal protocol for the fabrication of graphitized porous carbon materials and boost their performances in a broad range of applications including energy storage, catalysis, electroassisted separation, and others.

## Conflict of Interest

The authors declare no conflict of interest.

## Supporting information

Supporting Information

## Data Availability

Research data are not shared.
