# Supplementary material for: Molecular Engineering toward High‐Crystallinity Yet High‐Surface‐Area Porous Carbon Nanosheets for Enhanced Electrocatalytic Oxygen Reduction
Source: Adv Sci (Weinh). 2021 Nov 16;9(3):2103477. doi: 10.1002/advs.202103477 (PMC8787383; doi:10.1002/advs.202103477)
Supplement: Supplementary file 1 — Supporting Information [file ADVS-9-2103477-s001.pdf]

© 2021 Wiley-VCH GmbH

ADVANCED SCIENCE Open Access

Supporting Information

for *Adv. Sci.,* DOI: 10.1002/advs.202103477

Molecular Engineering toward High-Crystallinity Yet High-Surface-Area Porous Carbon Nanosheets for Enhanced Electrocatalytic Oxygen Reduction

Yongqi Chen, Junlong Huang, Zirun Chen, Chenguang Shi, Haozhen Yang, Youchen Tang, Zongheng Cen, Shaohong Liu, Ruowen Fu*, and Dingcai Wu*

Supporting Information

Molecular Engineering toward High-Crystallinity Yet High-Surface-Area Porous Carbon Nanosheets for Enhanced Electrocatalytic Oxygen Reduction

Yongqi Chen, Junlong Huang, Zirun Chen, Chenguang Shi, Haozhen Yang, Youchen Tang, Zongheng Cen, Shaohong Liu, Ruowen Fu*, and Dingcai Wu*

Experimental Section

Chemical reagents and materials. $FeCl_3 \cdot 6H_2O$, NaOH and HF were obtained from Aladdin. HCl was obtained from Guangzhou Chemical Reagent Factory. anilino-methyl-triethoxysilane, (3-aminopropyl)trimethoxysilane and aniline were obtained from MACKLIN. Unless otherwise stated, all the reagents were of analytical grade and were used as received. All aqueous solutions were prepared with DI water.

Synthesis of GPCNSs. Typically, anilino-methyl-triethoxysilane (AMS) and $FeCl_3 \cdot 6H_2O$ with a molar ratio of 1:5 were firstly mixed at 50 ^oC for 0.5 h , followed by ageing at room temperature for 1 h. The as-obtained *x*PAMS/$FeCl_3 \cdot 6H_2O$ mixture were then thermally annealed under flowing N_2 at 700 ^oC for 3 h with a ramp rate of 5 ^oC min^{-1}. After washing with 6 M HCl and 12 M HF in sequence, the pyrolyzed products were subjected to further graphitization at 1000 ^oC for 1 h, giving rise to the target products GPCNSs. The control experiments were also performed by annealing the *x*PAMS/$FeCl_3 \cdot 6H_2O$ mixture in flowing N_2 at 600 and 800 ^oC, respectively, followed by treatments under identical conditions. With aniline and (3-aminopropyl) trimethoxysilane as the precursors, GCNSs and PCNSs were prepared under identical conditions for the comparison, respectively.

Material characterization. The nanomorphologies were investigated by a scanning electron microscope (SEM, Hitachi S-4800), a transmission electron microscope (TEM, FEI Tecnai G2 F20 S-TWIN), and an atomic force microscopy (AFM, Shimadzu SPM-9500J3). FTIR spectra were provided by the Thermo NICOLET 6700. ^{13}C NMR spectra were collected on Bruker AVANCE 400. Raman measurements

were recorded on a HORIBA JY LabRAM HR Evolution with excitation laser beam wavelength of 532 nm. X-ray photoelectron spectra were measured on a Thermo Fisher Scientific K-Alpha (15 kV, 10 mA). Pore structures were conducted on a Micromeritics ASAP 2020 analyser at 77 K. Specific surface area and pore size distribution were calculated based on Brunauer-Emmett-Teller theory and original Density Function Theory, respectively. XRD patterns were obtained on a RIGAKU D-MAX 2200 VPC with Cu-Kα radiation (40 kV, 26 mA).

Calculation of graphitization degree. The graphitization index (g_p) can be used to quantitatively characterize the graphitization degree of carbon materials from XRD patterns according Equation (S1):

$$g_p = (0.3440 - d_{002}) / (0.3440 - 0.3354) \qquad \text{(S1)}$$

The crystalline structure parameters, including lateral size (L_a) and stacking height (L_c), can be estimated using Scherrer Equations (S2) and (S3):

$$L_a = 1.84\, \lambda / \beta_a \cos\theta_a \qquad \text{(S2)}$$

$$L_c = 0.89\, \lambda / \beta_c \cos\theta_c \qquad \text{(S3)}$$

where θ_a and θ_c is the diffraction angle of (110) and (002) crystal faces, and β_a and β_c is the full width at the half maximum (FWHM) of each peak, respectively. According to the Bragg's law, the interplanar spacing $d_{002} = \lambda / 2\sin(\theta_c)$, where λ (0.1541 nm) is the wavelength of Cu Kα.[1,2]

Electrochemical measurements. All the electrochemical measurements were carried out in a conventional three-electrode cell at room temperature using a CHI660E electrochemical workstation (Shanghai Chenhua Instruments Limited, China). Ag/AgCl and platinum plate electrode were used as reference and counter electrodes, respectively. RDE electrode with a 0.196 cm^2 glassy carbon disk as well as RRDE electrode with a Pt ring (6.5 mm inner-diameter and 8.5 mm outer-diameter) and a glassy carbon disk (5.5 mm diameter) were used as the working electrode for evaluating the ORR activity and selectivity of various catalysts. The electrochemical experiments were carried in O_2 saturated 0.1 M KOH electrolyte for ORR and 1 M KOH electrolyte for OER. The CV and LSV curves were recorded at a scan rate of 20

and 10 mV s^{-1}, respectively.

The catalyst ink was prepared by blending the catalyst powder (5 mg) with 50 μL Nafion solution (~5% in a mixture of lower aliphatic alcohols and water from Sigma-Aldrich), 500 μL ethanol and 450 μL water in an ultrasonic bath. A certain amount of catalyst ink was then pipetted onto the glassy carbon disk, leading to a catalyst loading of 0.5 mg cm^{-2} for all samples except Pt/C (20 wt%, 0.2 mg cm^{-2}). All potentials of electrochemical measurements were calibrated with respect to reversible hydrogen electrode (RHE) according to E (V vs. RHE) = E (V vs. Ag/AgCl) + 0.197 + 0.059pH.

The H_2O_2 yield can be calculated from the following Equation (S4) to evaluate the four-electron selectivity of catalysts, and the electron transfer number can be calculated based on the following Equation (S5):

$$H_2O_2\,\% = 2 \times (I_R / N) / (I_D + I_R / N) \times 100\% \qquad \text{(S4)}$$

$$n = 4 \times I_D / (I_D + I_R / \mathrm{N}) \qquad \text{(S5)}$$

here, I_R and I_D are the ring and disk currents, respectively, and N = 0.38 is the ring collection efficiency. [3]

Koutecky-Levich (K-L) plots were obtained according to the following Equation (S6):

$$1/J = 1/J_k + 1/B\omega^{1/2} \qquad \text{(S6)}$$

where J_k is the kinetic current at a constant potential and ω represents the electrode rotating speed. B can be determined from the slope of the K-L plots based on Levich Equation (S7):

$$B = 0.2\, n\, F(D_{O2})^{2/3} \upsilon^{-1/6} C_{O2} \qquad \text{(S7)}$$

where n represents the transferred electron number per oxygen molecule, F is Faraday constant (96485 C mol^{-1}), D_{O2} is the diffusion coefficient of O_2 (1.9×10^{-5} cm^2 s^{-1}), υ is the kinetic viscosity (υ = 0.01 cm^2 s^{-1}) and C_{O2} is the bulk concentration of O_2 (1.2×10^{-6} mol cm^{-3}). When expressing the rotation speed in rpm, the constant 0.2 is applied.

In Tafel plot, the kinetic current density (J_k) was calculated from the mass-transport

correction of RDE data by the followed Equation (S8):

$$J_k = (J \times J_L)/(J_L - J) \qquad (S8)$$

Electrochemically active surface area (ECSA) is reflected by the electrochemical double layer capacitance (C_{dl}) of the catalytically active surface, which was measured from the slope of the anodic current density versus scan rates graph. The C_{dl} is calculated by using Equation (S9):

$$i = v\, C_{dl} \qquad (S9)$$

where i is the measured anodic current density and v is the scan rate. The plot of i versus ν gives a straight line with C_{dl} as a slope.

Electrochemical impedance spectroscopy (EIS) tests were carried out at the open circuit potential with a frequency range from 10^{-1} Hz to 10^{5} Hz.[3,4]

Zn-air battery test. The primary Zn-air batteries were tested in homemade electrochemical cells, where catalysts loaded on a carbon paper with gas diffusion layer (catalysts loading 2 mg cm^{-2} for GPCNSs and 1 mg cm^{-2} for Pt/C) as the air cathode, a zinc foil as the anode, and 6 M KOH solution as the electrolyte. The rechargeable Zn-air batteries were tested using identical cell configuration except add $Zn(Ac)_2$ (0.2 M) into 6 M KOH electrolyte.[5,6]

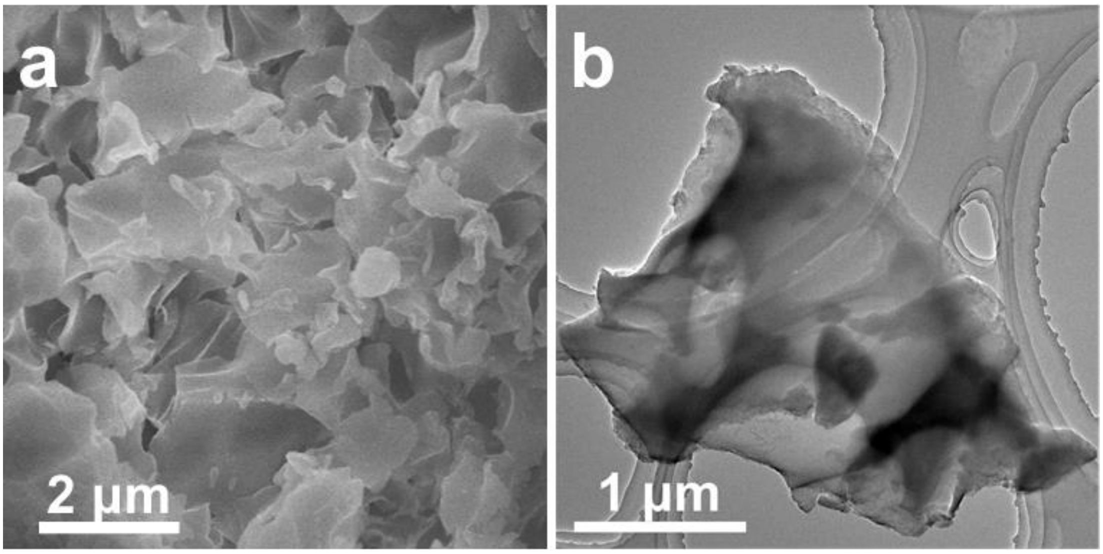

Figure S1. (a) SEM and (b) TEM images of *x*PAMS.

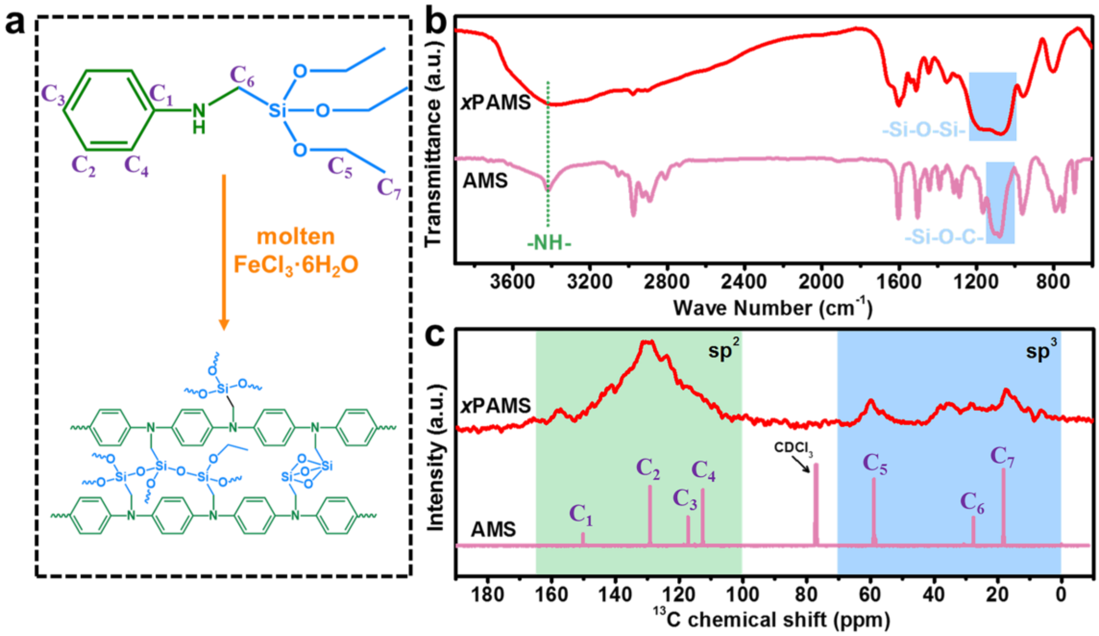

Figure S2. (a) Schematic illustration of the synthesis of *x*PAMS. (b) FT-IR spectra and (c) ^{13}C NMR spectra of *x*PAMS and AMS.

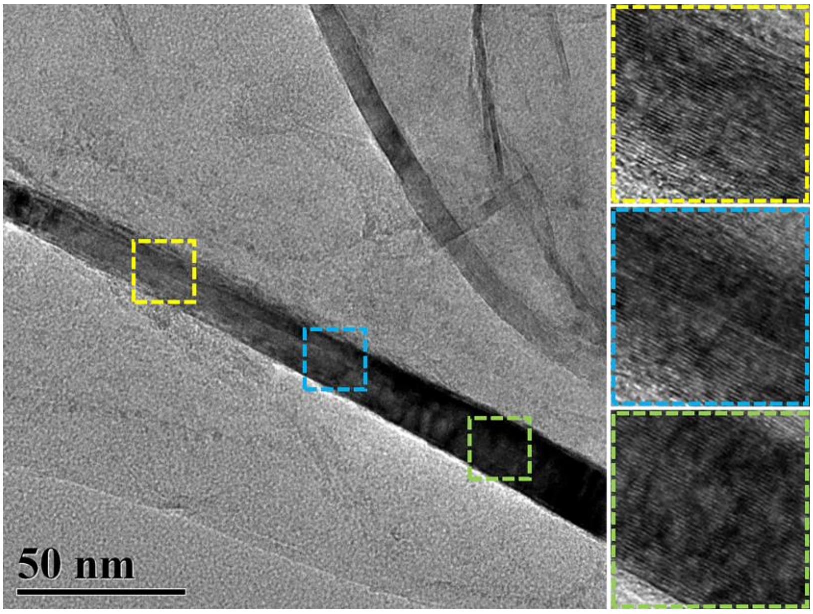

Figure S3. Large scope HRTEM images of GPCNSs to demonstrate the long-range order carbon layers.

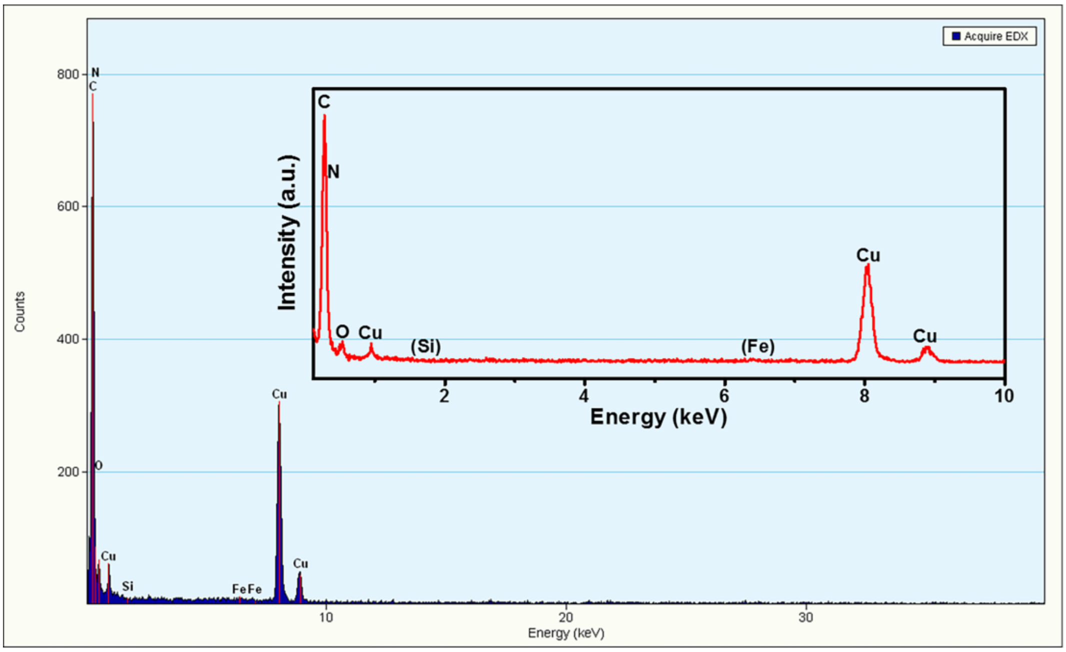

Figure S4. EDS spectrum of GPCNSs.

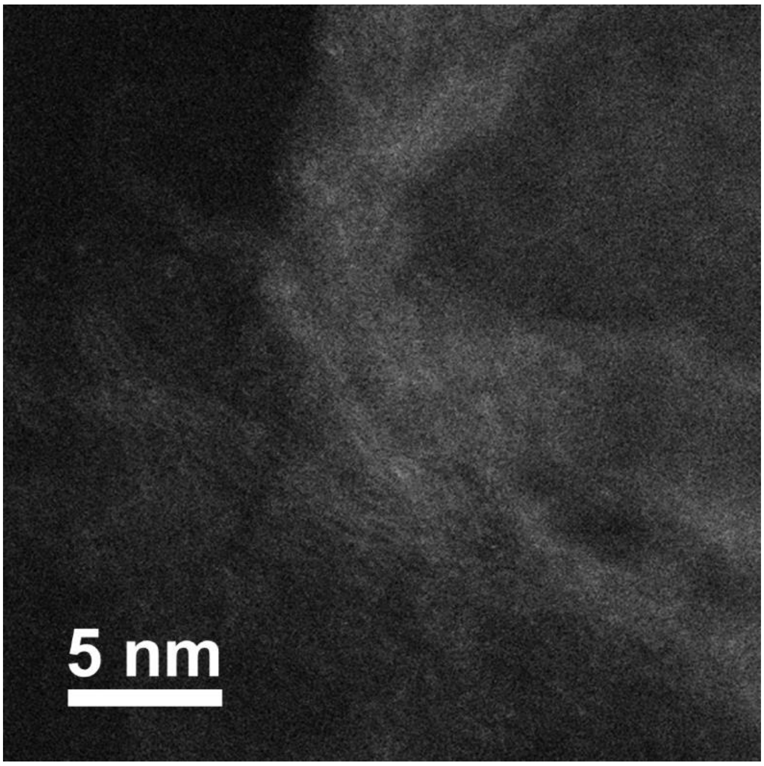

Figure S5. Aberration corrected HAADF-STEM image of GPCNSs.

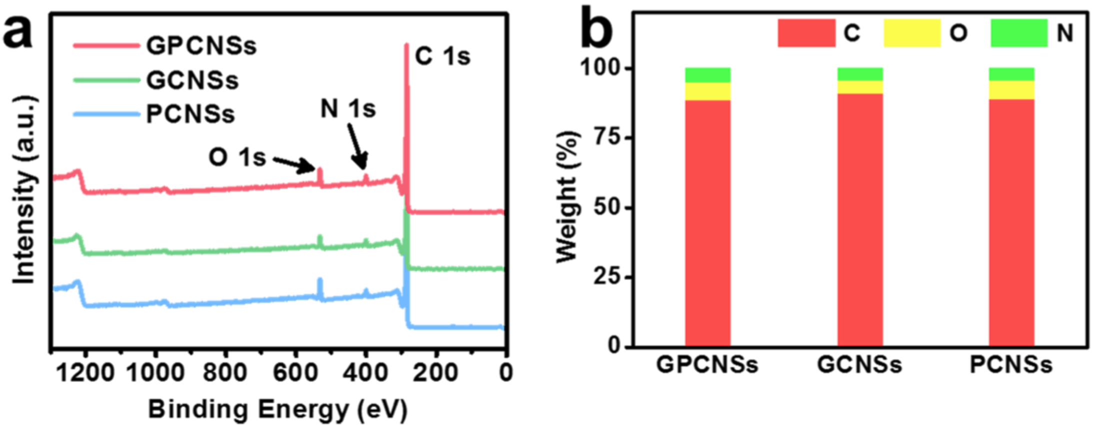

Figure S6. (a) XPS spectra and (b) the corresponding C, O and N contents of GPCNSs. The GPCNSs, GCNSs, and PCNSs exhibit similar N contents, which are 5.2 wt%, 4.8 wt%, and 4.5 wt%, respectively.

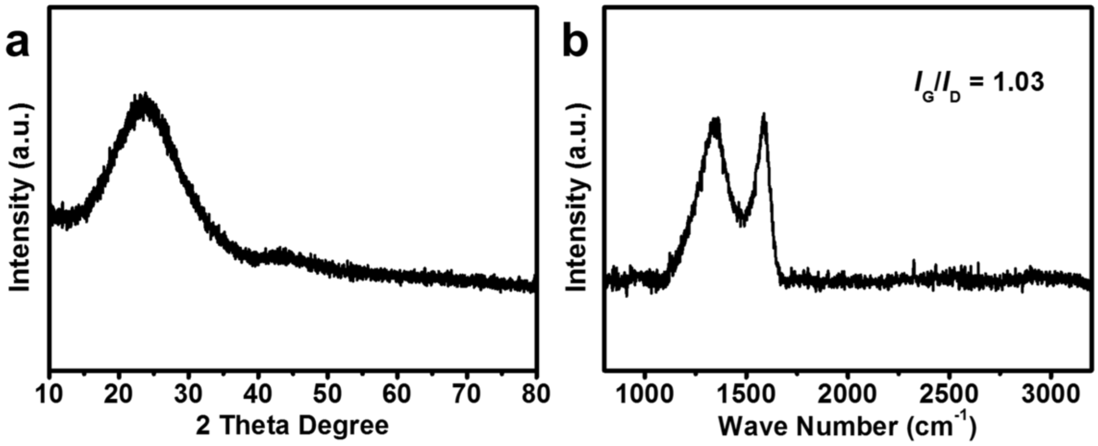

Figure S7. (a) XRD pattern and (b) Raman spectrum of the products obtained by pyrolyzing *x*PAMS without $FeCl_3 \cdot 6H_2O$ salt. The broad XRD peak at 23.7° with an I_G/I_D of 1.03 indicate a dominated amorphous structure.

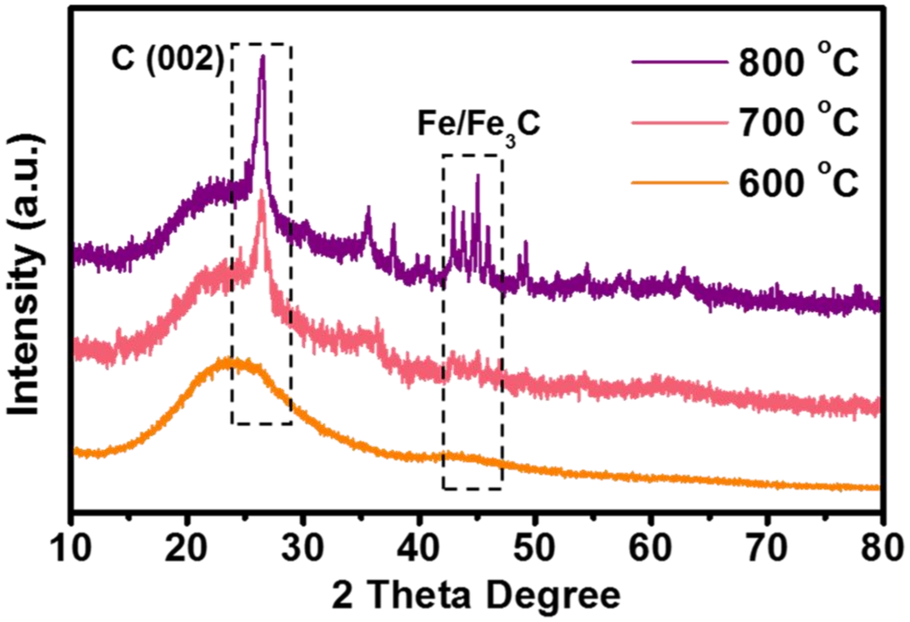

Figure S8. XRD patterns of *x*PAMS/$FeCl_3 \cdot 6H_2O$ mixture annealed at different temperatures without acid washing. The residue $FeCl_x \cdot yH_2O$ compound is firstly removed by water washing.

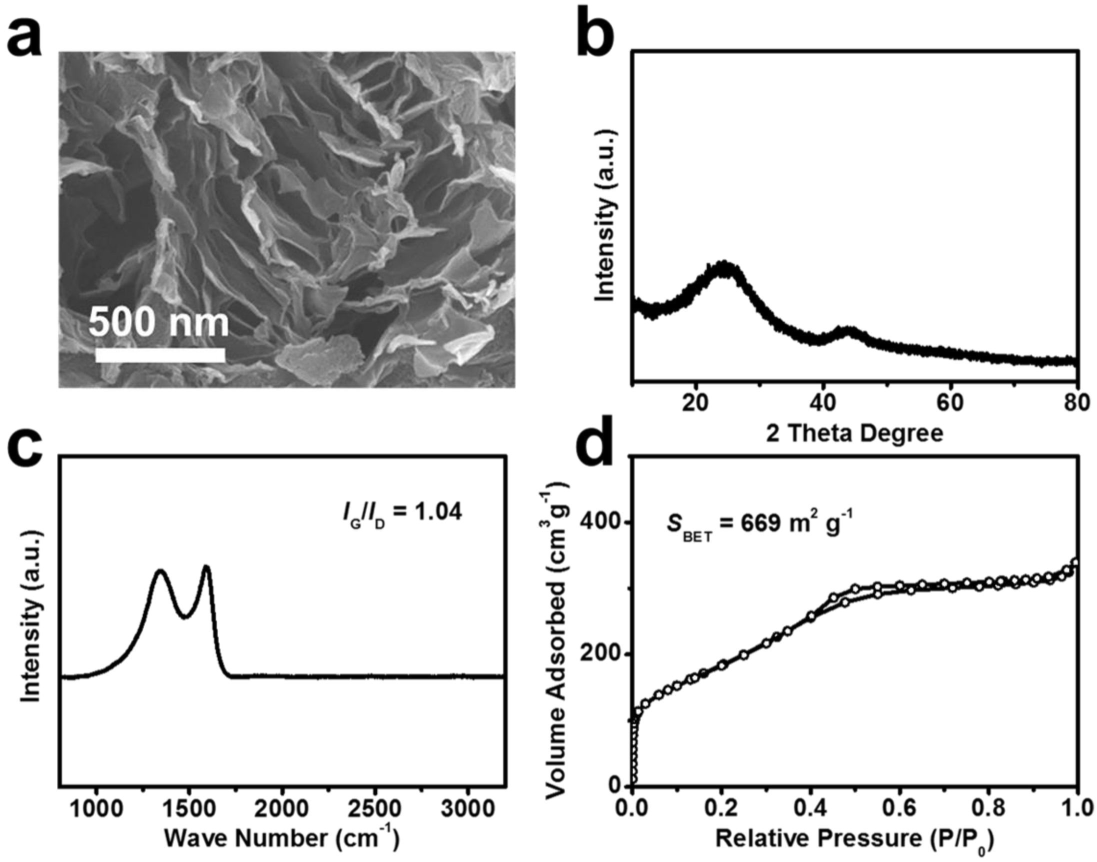

Figure S9. (a) SEM image, (b) XRD pattern, (c) Raman spectrum and (d) N_2 adsorption-desorption isotherm of the carbon nanosheets obtained by thermally annealing the *x*PAMS/$FeCl_3 \cdot 6H_2O$ mixture at 600 ^{o}C followed by graphitization treatment at 1000 ^{o}C.

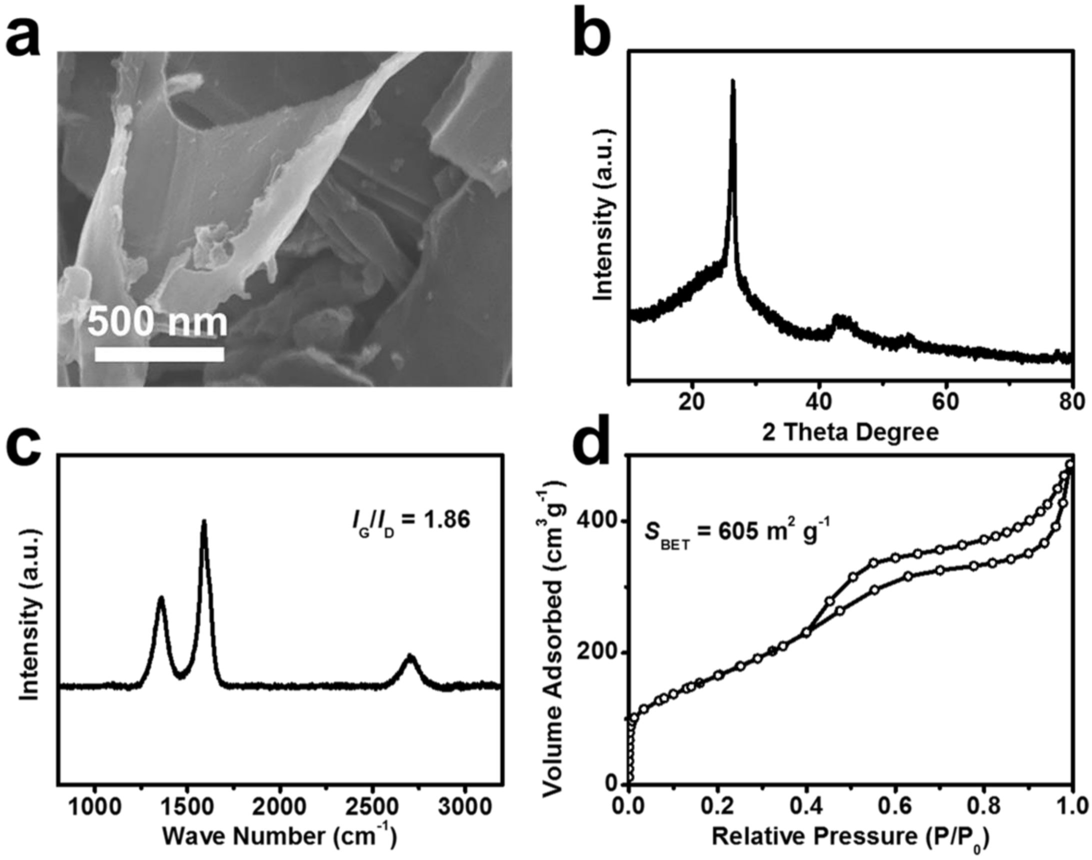

Figure S10. (a) SEM image, (b) XRD pattern, (c) Raman spectrum and (d) N_2 adsorption-desorption isotherm of the carbon nanosheets obtained by thermally annealing the *x*PAMS/$FeCl_3 \cdot 6H_2O$ mixture at 800 ^oC followed by graphitization treatment at 1000 ^oC.

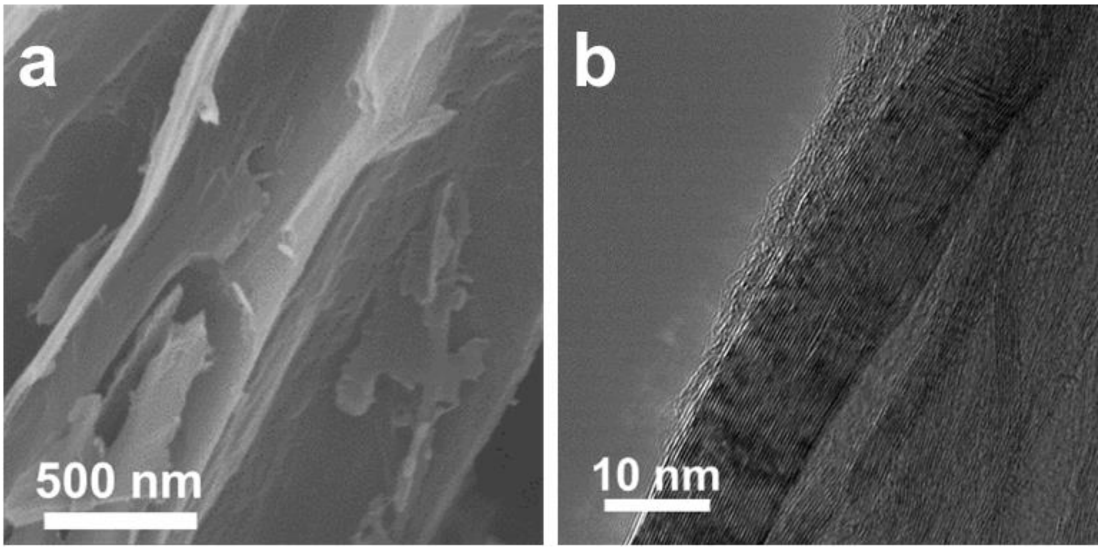

Figure S11. (a) SEM and (b) HRTEM images of GCNSs obtained from aniline precursor. The GCNSs are also composed of long-range order carbon layers with a well-defined interlayer spacing of 0.3373 nm, suggestive of highly graphitic structure.

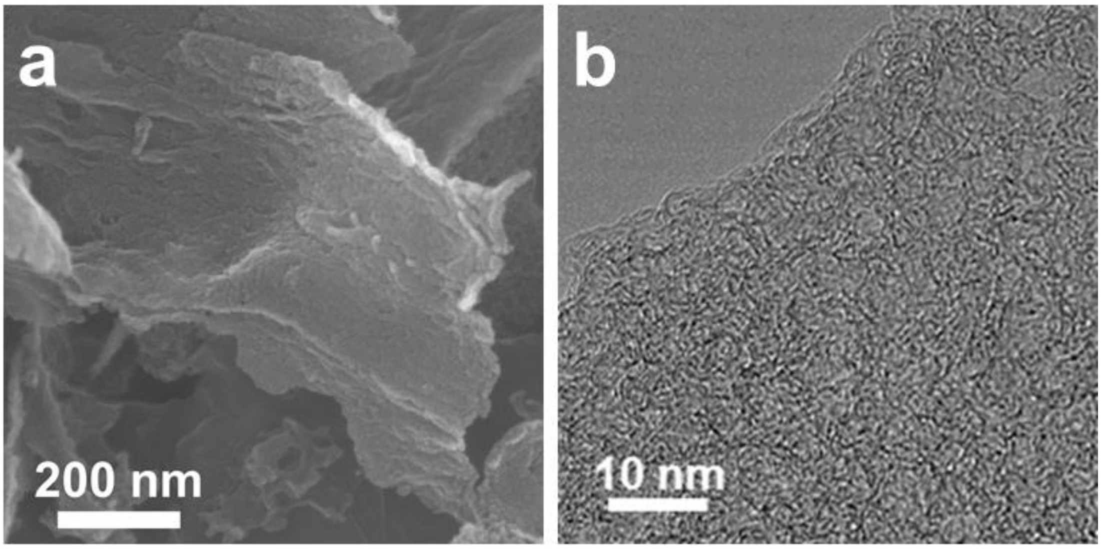

Figure S12. (a) SEM and (b) HRTEM images of PCNSs obtained from (3-aminopropyl) trimethoxysilane precursor. The PCNSs is composed of disorderly packed turbostratic carbon nanodomains.

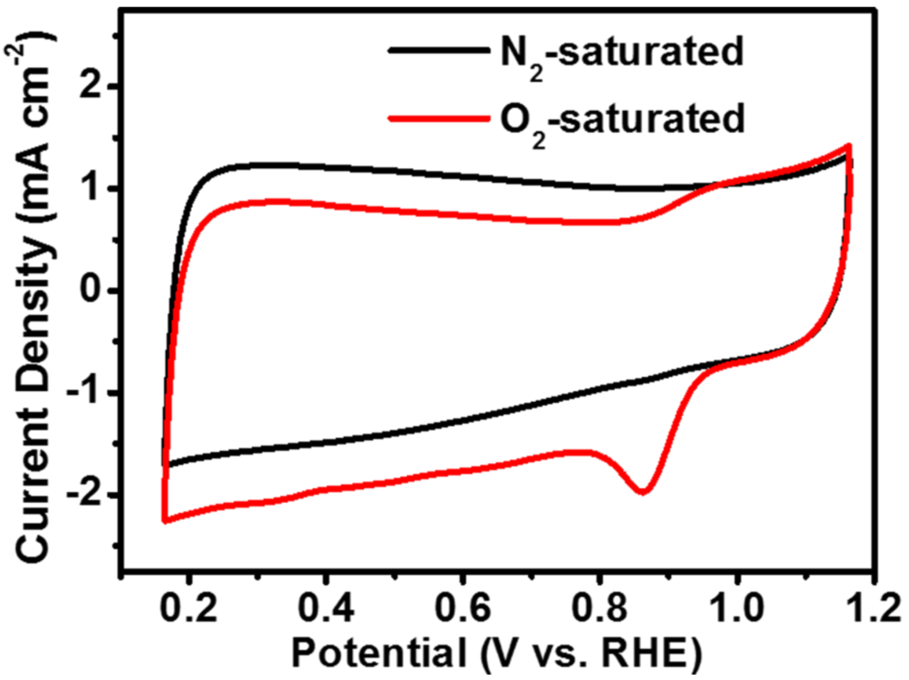

Figure S13. CV curves of GPCNSs conducted in N_2 or O_2-saturated 0.1 M KOH electrolyte at a scan rate of 20 mV s^{-1}.

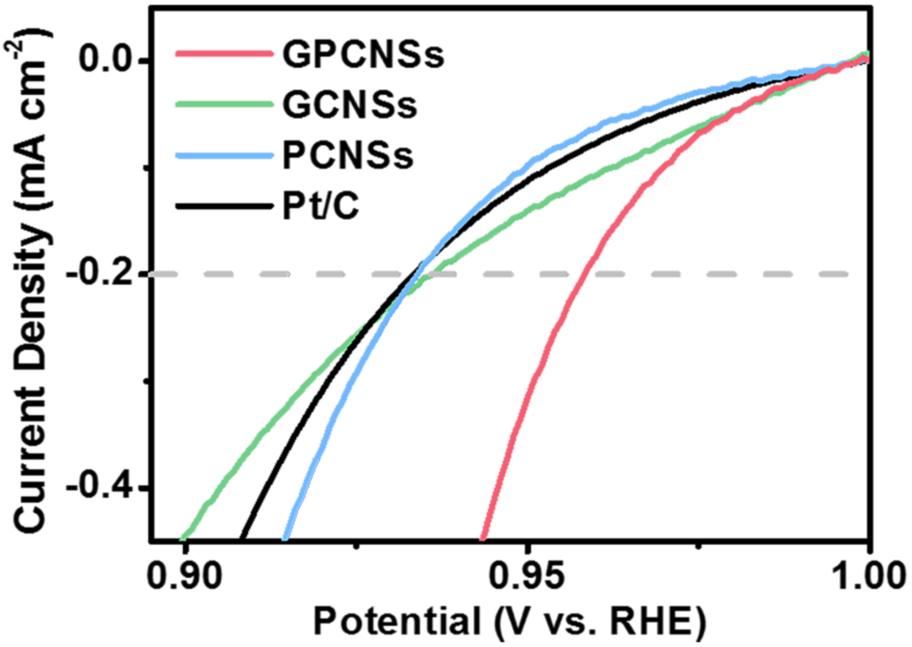

Figure S14. E_{onset} of GPCNSs, GCNSs, PCNSs and Pt/C, which are obtained depended on the potentials at a current density of 0.2 mA cm^{-2} current density.

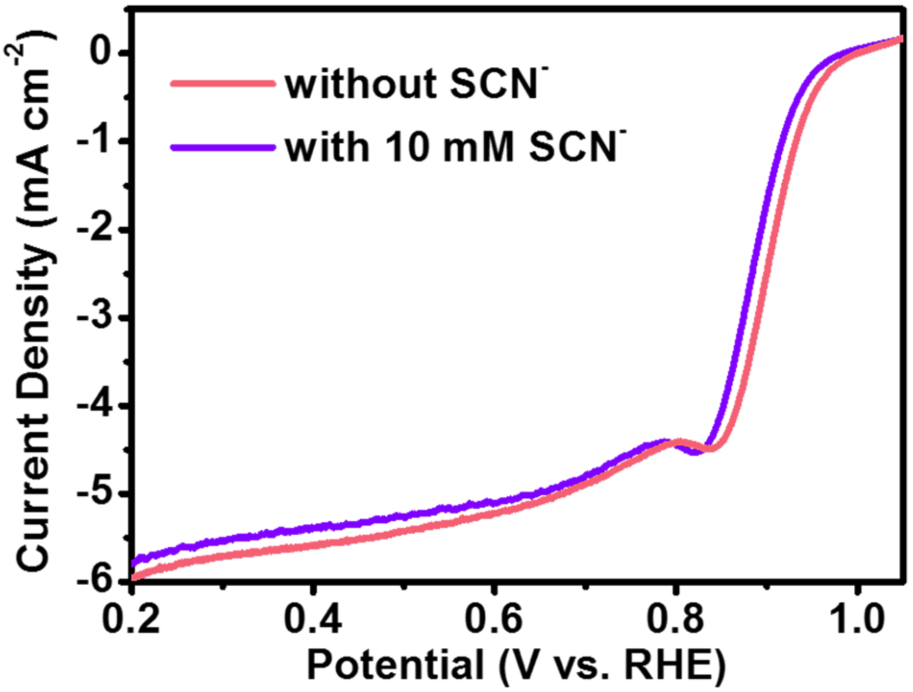

Figure S15. LSV curves of GPCNSs with/without 10 mM KSCN.

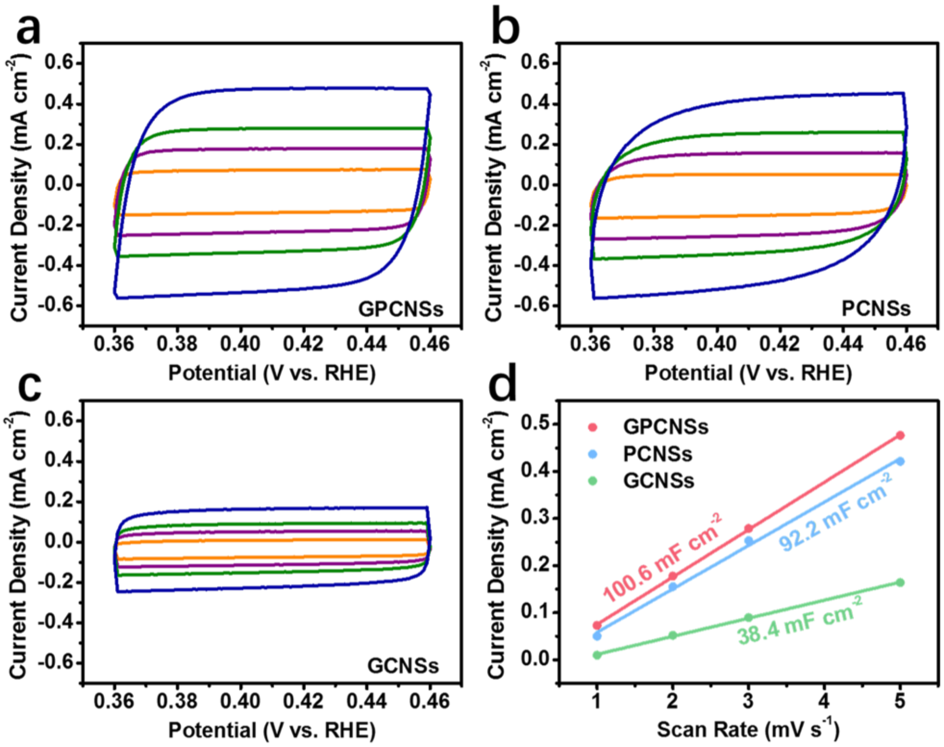

Figure S16. CV curves of (a) GPCNSs, (b) PCNSs, and (c) GCNSs tested at of scan rates of 1, 2, 3, and 5 mV s^{-1}, respectively. (d) Dependence of current density as a function of scan rates at 0.41 V.

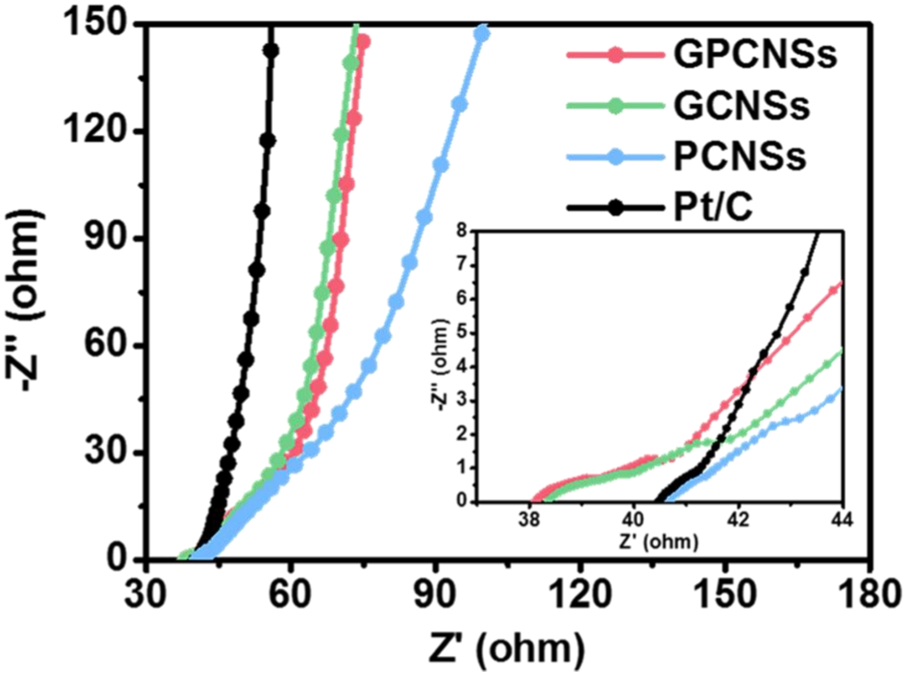

Figure S17. EIS plots of GPCNSs, GCNSs, PCNSs, and Pt/C catalysts. The inset image is the partially enlarged EIS plots.

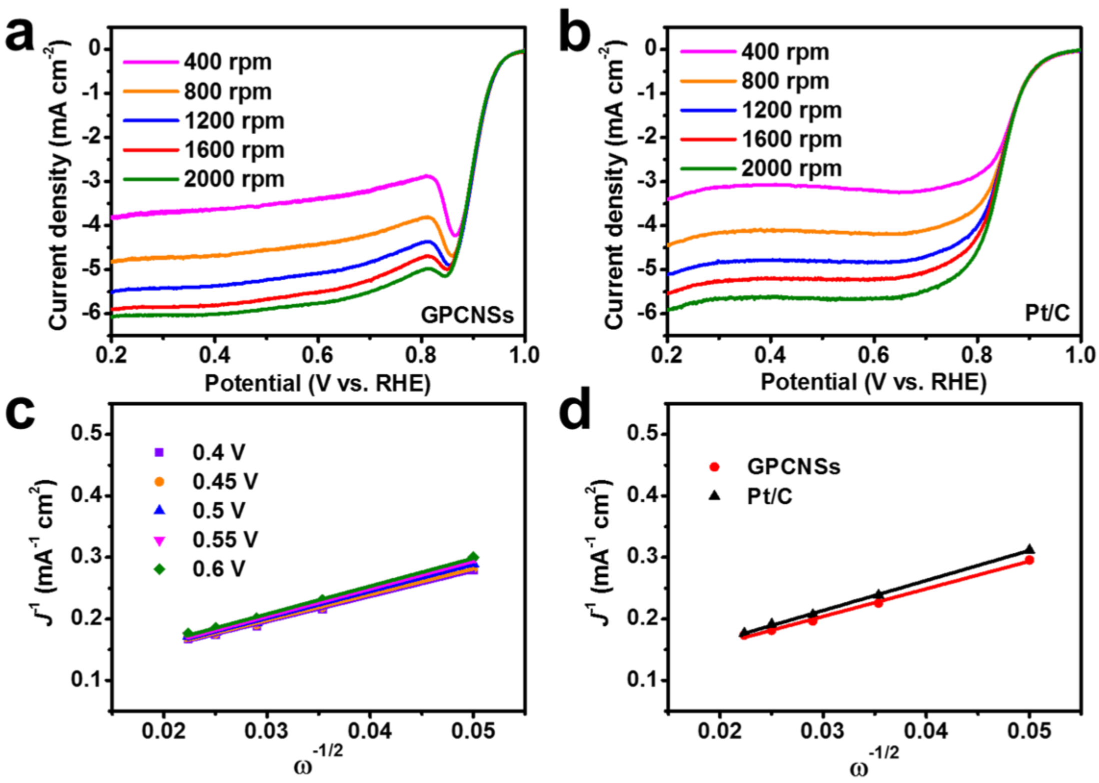

Figure S18. LSV curves of (a) GPCNSs and (b) Pt/C at different rotating speeds. K-L plots for (c) GPCNSs at various potential and for (d) GPCNSs and Pt/C at 0.6 V.

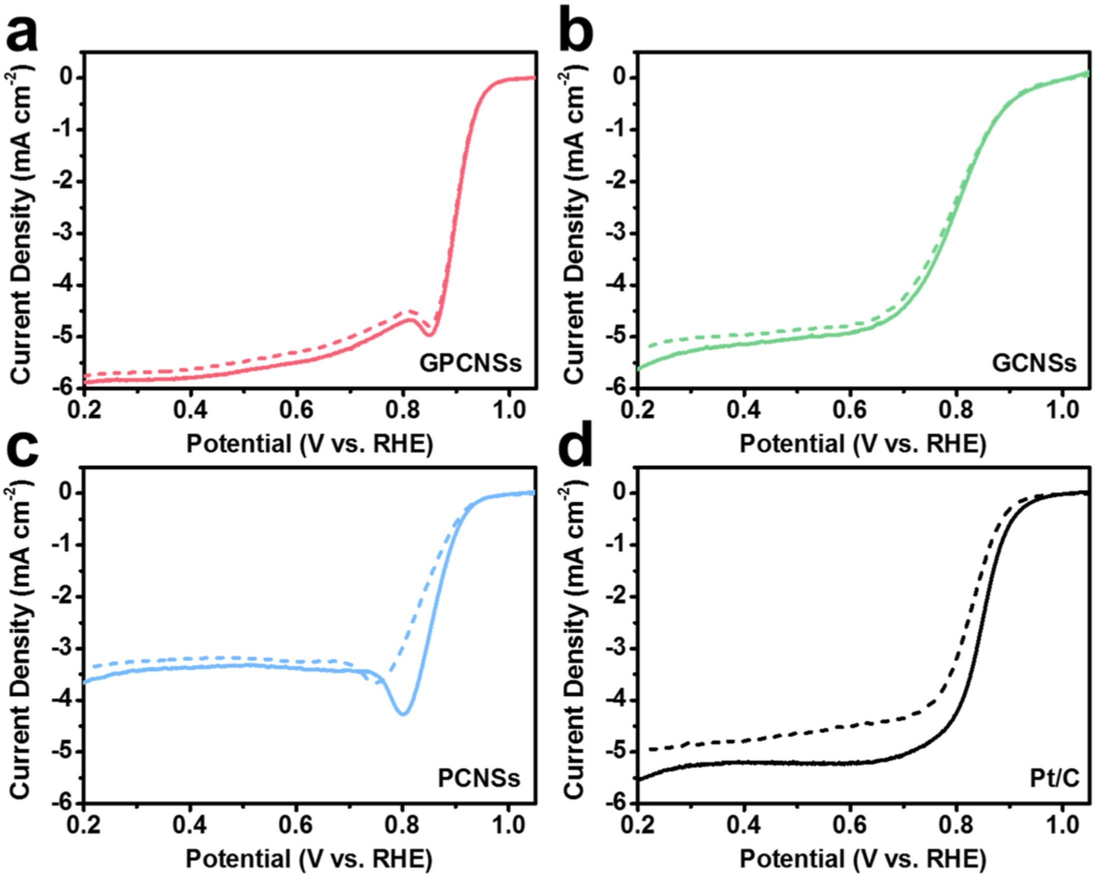

Figure S19. LSV curves of (a) GPCNSs, (b) GCNSs,(c) PCNSs and (d) Pt/C, before (solid line) and after (dash line) 5000 cycles in oxygen-saturated 0.1 M KOH in the potential range from 0.564 to 0.964 (V vs. RHE). GPCNSs and GCNSs show almost no potential shift of $E_{1/2}$, whereas PCNSs and Pt/C show visible potential shift.

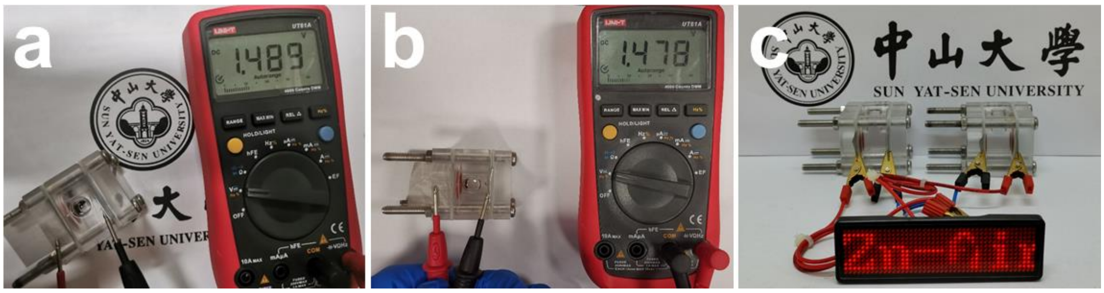

Figure S20. Open-circuit potential of the Zn-air batteries with (a) GPCNSs and (b) Pt/C catalysts. (c) Optical image of a LED panel lighted by two homemade Zn-air batteries connected in series with GPCNSs catalysts.

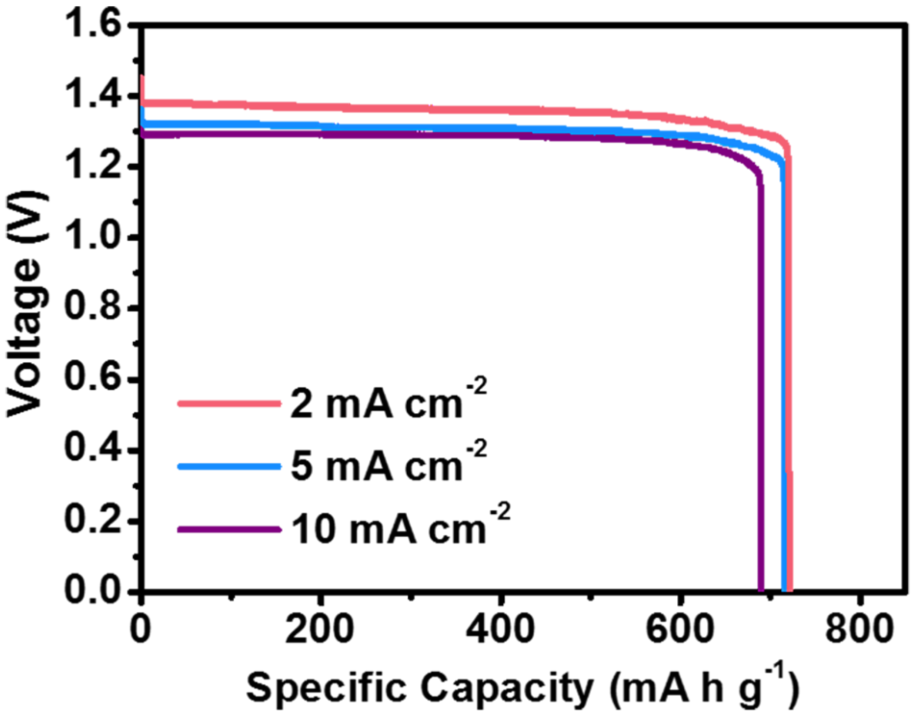

Figure S21. Galvanostatic discharge curves of Pt/C at different current densities. The specific capacity is normalized by the mass of the consumed Zn anode.

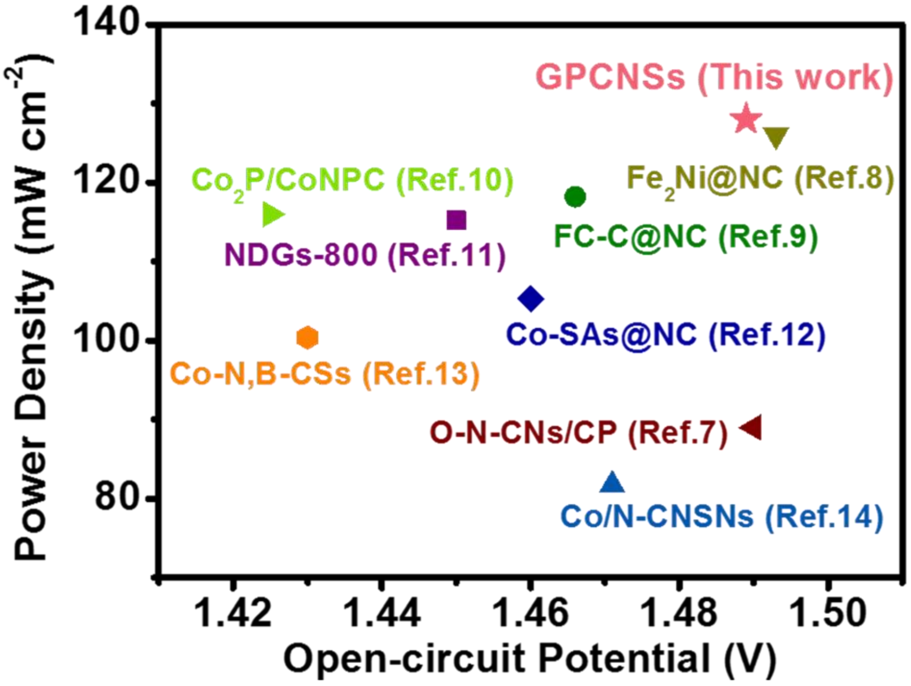

Figure S22. Performance comparison of Zn-air batteries between GPCNSs and other previously reported carbon-based catalysts.[7-14]

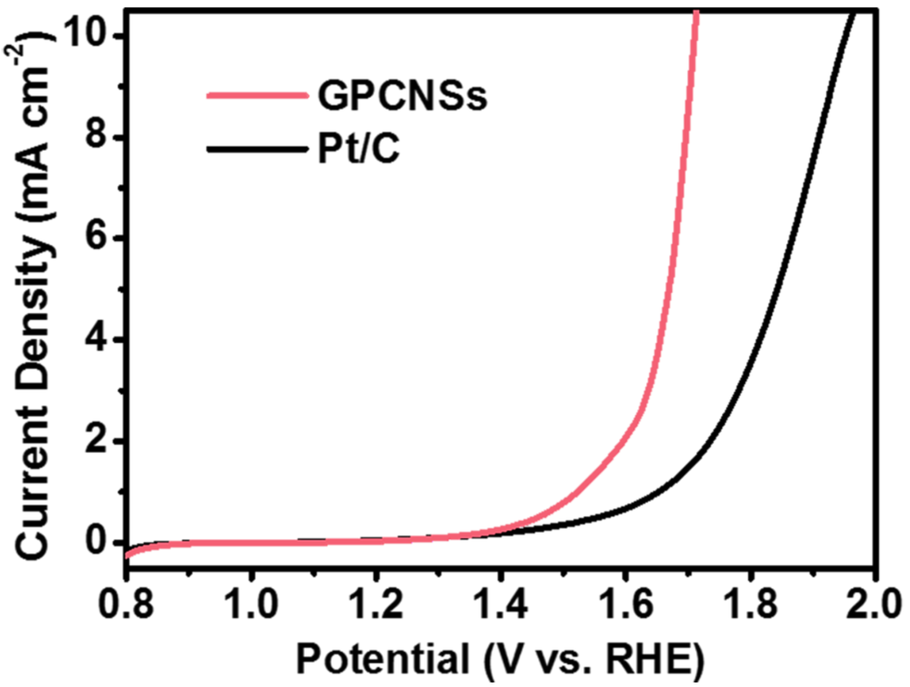

Figure S23. LSV curves of GPCNSs and Pt/C for OER.

Table S1 Structure parameters of GPCNSs, GCNSs, and PCNSs obtained from XRD patterns, Raman spectra, and N_2 adsorption-desorption isotherms.

**Samples**	**S_{BET} (m^2 g^{-1})**	**I_G/I_D**	**g_p***
GPCNSs	1342	1.84	0.76
GCNSs	663	1.87	0.78
PCNSs	1411	1.18	/

*g_p of PCNSs cannot be obtained because of unobservable (002) peak in XRD pattern.

Table S2. Summary of recently reported ORR performances of different carbon-based catalysts under alkaline conditions (0.1 M KOH).

**Samples**	**$E_{1/2}$ (V vs. RHE)**	**Reference**
**GPCNSs**	**0.897**	**This work**
**PCNSs**	**0.866**	**This work**
**GCNSs**	**0.783**	**This work**
Co-C_3N_4/CNT	0.86	J. Am. Chem. Soc. 2017, 139, 3336-3339
Fe/N-G-SAC	0.89	Adv. Mater. 2020, 32, 2004900
NDC1000	0.86	Angew. Chem. Int. Ed. 2020, 59, 11999-12006
OLC/Co-N-C	0.855	Angew. Chem. Int. Ed. 2021, 60, 12759-12764
CNT/PC	0.88	J. Am. Chem. Soc. 2016, 138, 15046
P-NCNS	0.889	Carbon 2021, 174, 404-412
Cu/Zn-NC	0.83	Angew. Chem. Int. Ed. 2021, 60, 14005-14012
Fe-N_4-C-60	0.80	Adv. Mater. 2020, 32, 2000966

N, P-GDs/N-3DG	0.81	ACS Appl. Mater. Interfaces 2021, 13, 30512-30523
Fe_{SA}/NSC	0.87	J. Mater. Chem. A 2021, 9, 10110-10119
a-MoC/NHPC	0.88	Energy Environ. Sci. 2020, 13, 2849-2855
Fe-SCNS	0.89	Angew. Chem. Int. Ed. 2020, 59, 19627-19632
10Co-N@DCNF	0.83	Angew. Chem. Int. Ed. 2020, 59, 6122-6127
MnNC-PDA-700	0.87	Energy Storage Mater. 2021, 37, 274-282

Supplementary References

[1] H. Wang, S. Min, C. Ma, Z. Liu, W. Zhang, Q. Wang, D. Li, Y. Li, S. Turner, Y. Han, H. Zhu, E. Abou-hamad, M. N. Hedhili, J. Pan, W. Yu, K. W. Huang, L. J. Li, J. Yuan, M. Antonietti, T. Wu, *Nat. Commun.* **2017**, 8, 13592.

[2] S. Zhang, Q. Liu, H. Zhang, R. Ma, K. Li, Y. Wu, B. J. Teppen, *Carbon* **2020**, 157, 714.

[3] K. Wu, L. Zhang, Y. Yuan, L. Zhong, Z. Chen, X. Chi, H. Lu, Z. Chen, R. Zou, T. Li, C. Jiang, Y. Chen, X. Peng, J. Lu, *Adv. Mater.* **2020**, 32, 2002292.

[4] S. Nandi, S. K. Singh, D. Mullangi, R. Illathvalappil, L. George, C. P. Vinod, S. Kurungot, R. Vaidhyanathan, *Adv. Energy Mater.* **2016**, 6, 1601189.

[5] S. Li, C. Cheng, H. W. Liang, X. Feng, A. Thomas, *Adv. Mater.* **2017**, 29, 1700707.

[6] S. Liu, Z. Wang, S. Zhou, F. Yu, M. Yu, C. Y. Chiang, W. Zhou, J. Zhao, J. Qiu, *Adv. Mater.* **2017**, 29, 1700874.

[7] J. J. Lv, Y. Li, S. Wu, H. Fang, L. L. Li, R. B. Song, J. Ma, J. J. Zhu, *ACS Appl. Mater. Interfaces* **2018**, 10, 11678.

[8] J. Zhu, M. Xiao, G. Li, S. Li, J. Zhang, G. Liu, L. Ma, T. Wu, J. Lu, A. Yu, D. Su, H. Jin, S. Wang, Z. Chen, *Adv. Energy Mater.* **2019**, 10, 1903003.

[9] K. Zhang, Y. Zhang, Q. Zhang, Z. Liang, L. Gu, W. Guo, B. Zhu, S. Guo, R. Zou, *Carbon Energy* **2020**, 2, 283.
[10] H. Liu, J. Guan, S. Yang, Y. Yu, R. Shao, Z. Zhang, M. Dou, F. Wang, Q. Xu, *Adv. Mater.* **2020**, 32, 2003649.
[11] Q. Wang, Y. Ji, Y. Lei, Y. Wang, Y. Wang, Y. Li, S. Wang, *ACS Energy Lett.* **2018**, 3, 1183.
[12] X. Han, X. Ling, Y. Wang, T. Ma, C. Zhong, W. Hu, Y. Deng, *Angew. Chem. Int. Ed.* **2019**, 58, 5359.
[13] Y. Guo, P. Yuan, J. Zhang, Y. Hu, I. S. Amiinu, X. Wang, J. Zhou, H. Xia, Z. Song, Q. Xu, S. Mu, *ACS Nano* **2018**, 12, 1894.
[14] X. Huang, Y. Zhang, H. Shen, W. Li, T. Shen, Z. Ali, T. Tang, S. Guo, Q. Sun, Y. Hou, *ACS Energy Lett.* **2018**, 3, 2914.
